# PAX2 maintains the differentiation of mouse oviductal epithelium and inhibits the transition to a stem cell-like state

**DOI:** 10.18632/oncotarget.20173

**Published:** 2017-08-10

**Authors:** Kholoud Alwosaibai, Atefeh Abedini, Ensaf M. Al-Hujaily, Yong Tang, Kenneth Garson, Olga Collins, Barbara C. Vanderhyden

**Affiliations:** ^1^ Department of Cellular and Molecular Medicine, University of Ottawa, Ontario, Canada; ^2^ Cancer Therapeutics Program, Ottawa Hospital Research Institute, Ontario, Canada; ^3^ King Fahad Specialist Hospital, Dammam, Saudi Arabia; ^4^ Department of Urology, Affiliated Cancer Hospital of Guangxi Medical University, Nanning, China; ^5^ Department of Obstetrics and Gynecology, University of Ottawa, Ontario, Canada

**Keywords:** fallopian tube, ovarian cancer, stem cells, epithelial-mesenchymal transition, PAX2

## Abstract

Recent studies have provided evidence that the secretory cells of the fallopian tube (oviduct) are a probable origin for high-grade serous ovarian carcinoma. In addition to secretory cells, the fallopian tube epithelium consists of ciliated cells and CD44+ undifferentiated stem-like cells. Loss of PAX2 expression is recognized as an early event in epithelial transformation, but the specific role of PAX2 in this transition is unknown. The aim of this study was to define the role of PAX2 in oviductal epithelial (OVE) cells and its response to transforming growth factor β1 (TGFβ), characterizing specifically its potential involvement in regulating stem cell-like behaviors that may contribute to formation of cancer-initiating cells. Treatment of primary cultures of mouse OVE cells with TGFβ induced an epithelial-mesenchymal transition (EMT) associated with decreased expression of PAX2 and an increase in the fraction of cells expressing CD44. PAX2 knockdown in OVE cells and overexpression in ovarian epithelial cells confirmed that PAX2 inhibits stem cell characteristics and regulates the degree of epithelial differentiation of OVE cells. These results suggest that loss of PAX2, as occurs in serous tubal intraepithelial carcinomas, may shift secretory cells to a more mesenchymal phenotype associated with stem-like features.

## INTRODUCTION

For more than 30 years, scientists focused on ovarian surface epithelial (OSE) cells and inclusion cysts derived from them as the origin of ovarian carcinomas [1, 2]. Researchers investigated factors that could contribute to the initiation of ovarian cancer, with the goal to identify mechanisms that inhibit this process [3, 4]. However, recent studies have provided evidence that fallopian tube epithelial cells are a probable cell of origin for many high-grade serous cancers [5–8], with precursor lesions frequently detected in the distal end (fimbria) of the fallopian tube in patients with a high risk for ovarian cancer [9].

Crum *et al*. have proposed a model to explain tumor progression from serous tubal intraepithelial carcinoma (STIC) to invasive carcinoma [10, 11], implicating secretory cell outgrowths (SCOUTs) as potential early precursor lesions. SCOUTs are regions of the fallopian tube epithelium in which expansion of secretory cells leads to regions of the epithelium consisting solely of secretory cells with the notable absence of ciliated cells. SCOUTs show frequent loss of paired box protein 2 (PAX2) expression and often acquire P53 expression [12]. PAX2 is a transcription factor that plays an important role during embryogenesis and development [13] and is required for fallopian tube and kidney formation [14], where it protects cells from apoptosis [15]. In adult tissues, PAX2 protein is present in normal oviductal epithelial cells (OVE), but it is not expressed in normal OSE [12]. A recent study reported that PAX2 is expressed in 100% of serous ovarian carcinoma cases [16]; however, this is not supported by previous studies which reported that as low as 9% of these cancers express PAX2 [17, 18]. The loss of PAX2 expression in SCOUTs and, although still controversial, its frequent loss in high-grade serous ovarian cancers underpin the need to better understand the biological cause and consequences of loss of PAX2 expression in OVE cells.

The origins of the cancer-initiating cells in STICs remain unclear, but tumor-initiating cells that are CD44+ have been isolated from mammary epithelial cells [19, 20] and colorectal cancer cells [21] and characterized as stem cells. Recent studies have reported putative stem cells located adjacent to the basement membrane of fallopian tube epithelium [22–24]. These basal cells are undifferentiated, possess self-renewal capacity, are CD44 positive, and do not express the markers of either secretory or ciliated cells. Interestingly, one study reported an expansion of CD44-positive cells in STICs compared to normal epithelium [22]. Another stem cell marker, ALDH1, was also found to be overexpressed in both STICs and putative basal stem cells [25], suggesting a possible origin of STICs from basal cells that possess stem cell-like activity. The importance of these markers in defining the role of stem cells in the progression from STICs to high-grade cancers remains controversial [26].

Early neoplasia in epithelial cells is often associated with loss of E-cadherin expression and an epithelial-mesenchymal transition (EMT). It has been hypothesized that epithelial cells undergoing EMT could include cells with stem cell functions [26], and stem-like cells in mouse and human breast carcinomas co-express EMT markers and stem cell markers [27]. While there is no evidence that OVE cells, SCOUTs or STICs undergo EMT, benign and malignant tumors are known to express EMT markers [28, 29]. Cells commonly respond to tissue wounding by activating EMT to refurbish the tissue [30]. In the ovary, ovulatory rupture activates OSE cells to acquire more mesenchymal characteristics to regenerate the cell layer [1], and EMT has been associated with increased stem-like characteristics in these cells [31]. The possibility that ovulation may also impact the OVE has been demonstrated by King and coworkers, who have shown increasing DNA damage in OVE in response to ovulation-associated inflammation [32]. Follicular fluid released at ovulation contains transforming growth factor β (TGFβ) [31], and Zhao and coworkers have found that fallopian tube epithelial cells express TGFβ ligands and receptors [33]. TGFβ induces EMT in many types of cells *in vitro*, including human ovarian epithelial and adenosarcoma cells, by enhancing *Snai1* (Snail) expression leading to inhibition of *Cdh1* (E-cadherin) [34, 35].

The aim of this study was to define the role of PAX2 in OVE cells, characterizing specifically its potential involvement in the regulation of stem cell-like behaviors that may be relevant to cancer-initiating cells. STICs are thought to arise from fallopian tube cell outgrowths that frequently have loss of PAX2 expression and show expansion of CD44 positive cells, and we herein provide evidence that knockdown of *Pax2* in OVE cells increases the expression of stem cell markers, increases the fraction of cells expressing CD44, and suppresses features of epithelial differentiation, all characteristics that could increase their susceptibility to tumor formation. Exposure of OVE cells to TGFβ suppresses *Pax2* expression, and elicits all of the same responses as *Pax2* knockdown. The ability of PAX2 to suppress stem cell characteristics was further confirmed in ovarian epithelial cells.

## RESULTS

### TGFβ induces EMT in OVE cells

**Figure 1:**
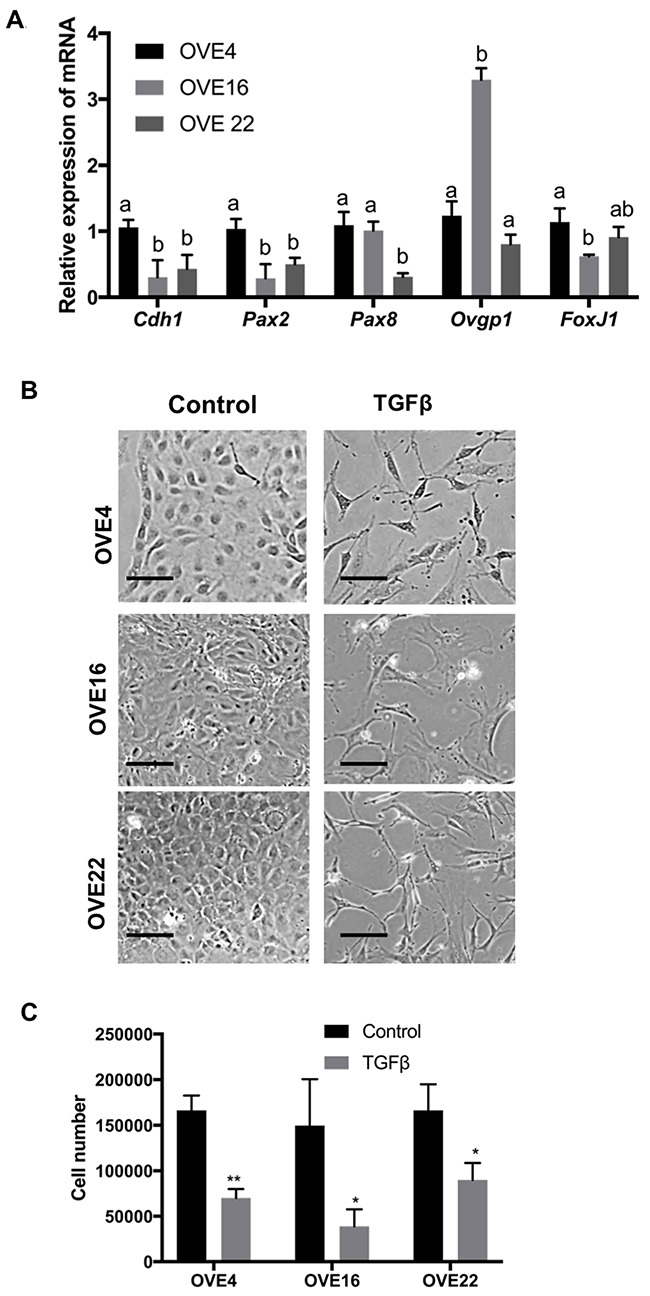
Characterization of clonal lines of oviductal epithelial cells **(A)** OVE4, OVE16 and OVE22 cells express oviductal cell markers (*Cdh1*, *Pax2*, *Pax8*, *Ovgp1* and *FoxJ1*). **(B)** TGFβ treatment (10ng/ml) for 7 days induced fibroblast-like phenotypes in OVE clonal cell lines and **(C)** inhibited cell proliferation in all OVE lines, as assessed after 7 days. Data presented in histograms are mean ± SEM. Scale bars indicate 100μm. All experiments were performed at least three times. In A, different letters denote values that are significantly different (p<0.05). In C, * indicates p<0.05; ** indicates p<0.01.

OVE cells were isolated from mouse oviducts and clonally grown into independent cell lines. The OVE clones have slightly different morphologies that reflect the varied expression of epithelial and OVE markers. Characterization of three clones is shown; OVE4 cells have an epithelial morphology (Figure 1B) and express the epithelial marker E-cadherin as well as the OVE markers PAX2, PAX8, OVGP and FoxJ1 (Figure 1A). OVE22 and OVE16 have mixed epithelial and mesenchymal morphologies (Figure 1B) and they express both epithelial and OVE markers. Notably, *Pax2* levels in OVE22 and OVE16 are lower than in OVE4 cells, and *Pax8* expression is much lower in OVE22.

**Figure 2:**
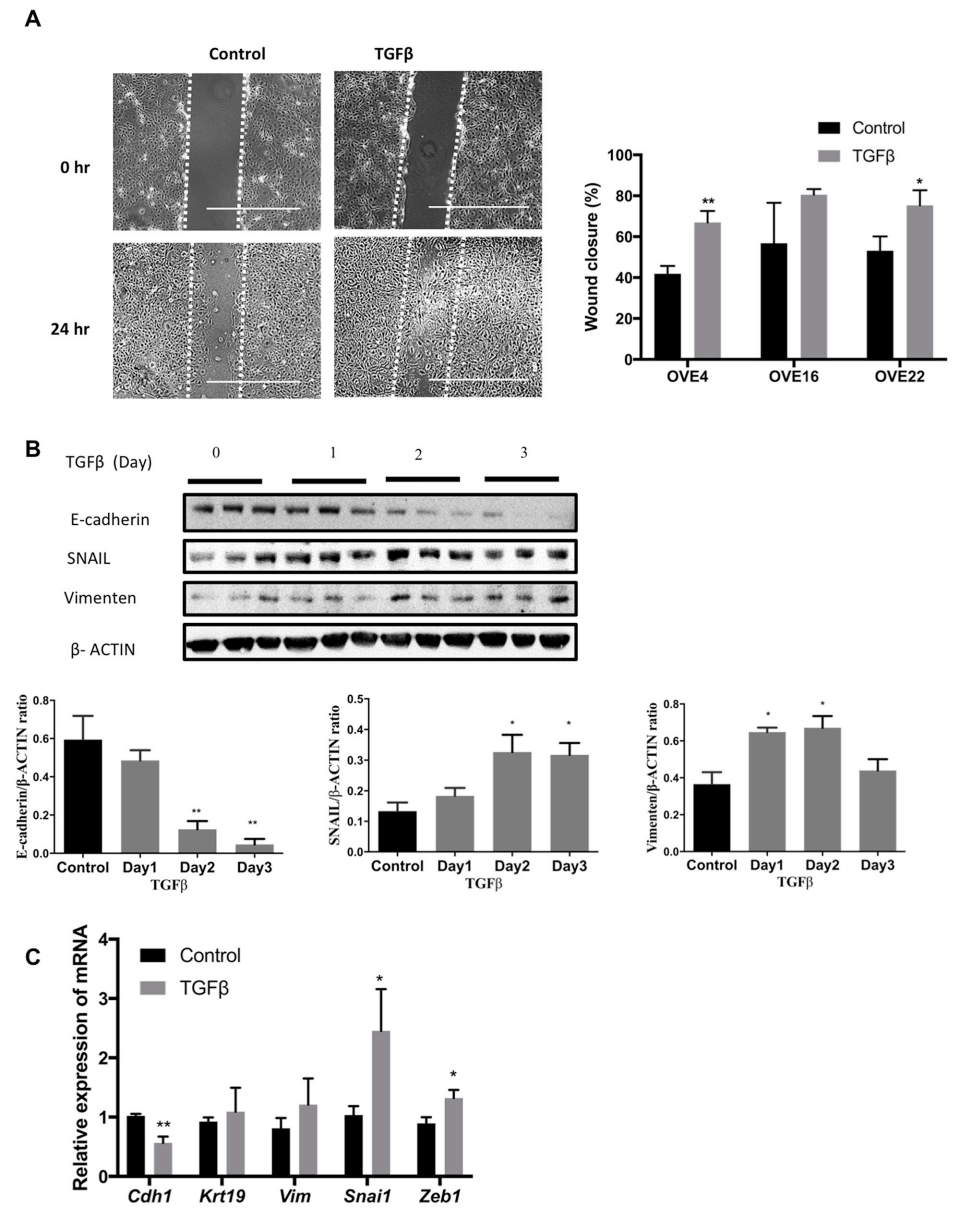
TGFβ induces EMT in oviductal epithelial cells **(A)** TGFβ pre-treatment for 3 days increased cell migration of OVE4 and OVE22 cells, as seen 24 hours after wounding. **(B)** Western blot and densitometric analysis for epithelial and EMT proteins (E-cadherin, SNAIL and Vimentin) in OVE4 cells treated with TGFβ for up to 3 days. **(C)** qPCR analysis for mRNA encoding for epithelial and EMT genes for OVE4 cells treated for one day with TGFβ. Data are from three independent experiments and are presented in histograms as mean ± SEM. Scale bars indicate 400μm. * indicates p<0.05; ** indicates p<0.01.

TGFβ treatment of all OVE clonal cells for seven days changed their morphology to more spindle-like cells (Figure 1B) and inhibited their proliferation (Figure 1C) without changes in their viability. Viability of both untreated and treated cells was at least 98% after four days of TGFβ treatment. Further investigation of this apparent EMT showed that TGFβ increased cell migration into ‘wounds’ created in monolayers of the OVE cells, although the results with OVE16 cells did not reach significance (Figure 2A). TGFβ treatment of OVE4 and OVE16 cells increased mRNA and protein levels of the EMT-associated transcription factor Snail and decreased E-cadherin expression (Figure 2B and 2C and Supplementary Figure 1). Given the similarities in their responses, all subsequent experiments were performed on OVE4 and OVE16 cells.

### TGFβ increases expression of stem cell markers in OVE cells

**Figure 3:**
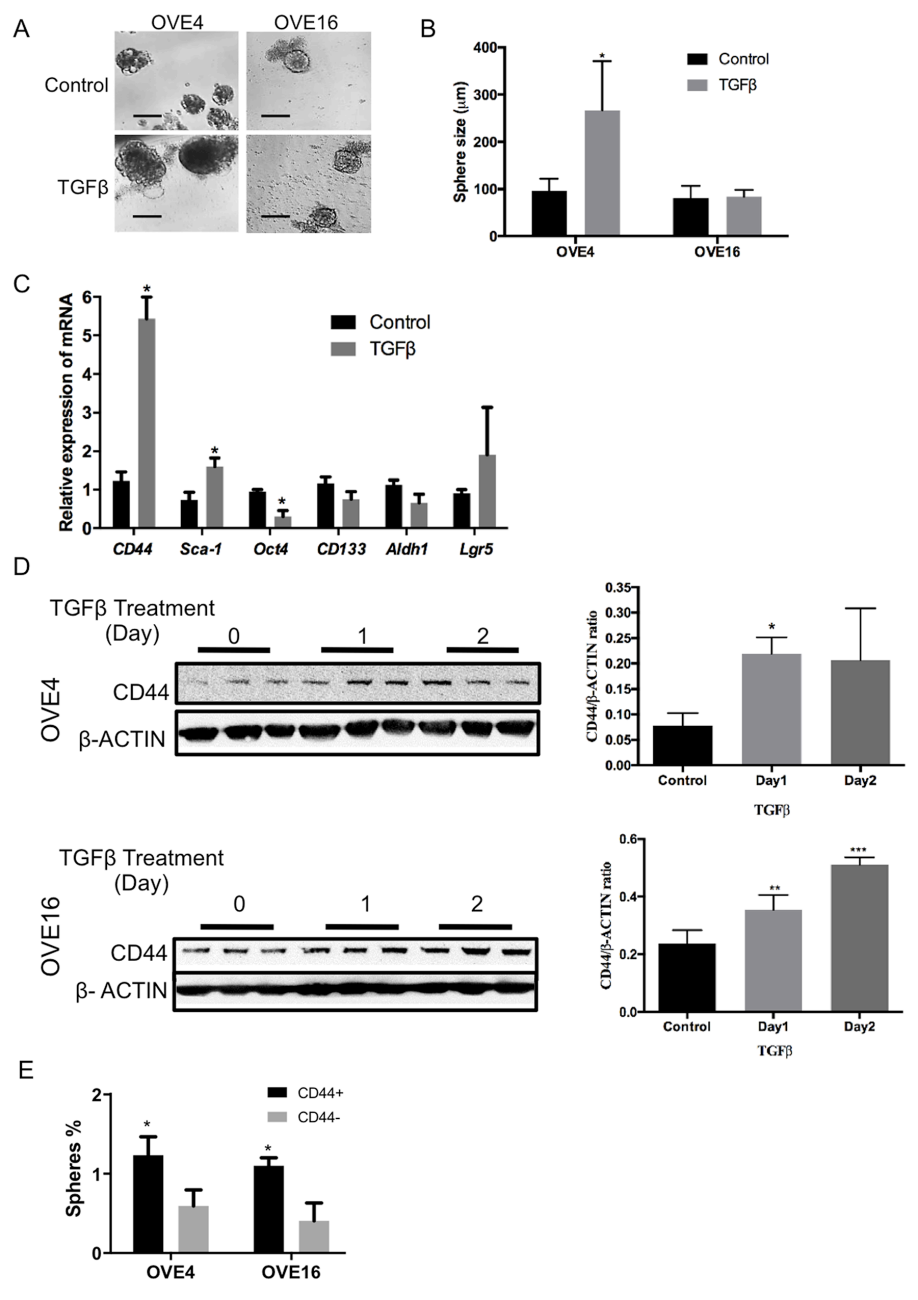
TGFβ increases the expression of stem cell markers in oviductal epithelial cells **(A and B)** OVE cells form spheres in low attachment plates and average sphere size is increased in OVE4 cells by TGFβ treatment. **(C)** Relative expression of mRNA encoding for stem cell markers in OVE4 shows that TGFβ treatment for 7 days significantly up-regulates *CD44* and, to a lesser extent, *Sca1* mRNA. **(D)** Western blots and densitometric analysis of those blots normalized to β-actin show increased expression of CD44 in OVE4 and OVE16 after 1 day of TGFβ treatment. **(E)** Sphere formation capacity of CD44 positive and negative populations sorted by flow cytometry. All data are from three independent experiments, Data presented in histograms are mean ± SEM. Scale bar in (A) is 100μm. * indicates p<0.05; ** p<0.01; *** p<0.001.

To determine if any of the OVE clonal cells possess stem cell characteristics, sphere formation assays were performed in low attachment plates with OVE cells treated with or without 10 ng/ml TGFβ for two weeks. For each OVE clonal cell line, a subset of the cells (~0.3%) was able to self-renew and form spheres in suspension (Figure 3A). TGFβ had no effect on the number of spheres (data not shown), but significantly increased the size of the spheres made by the OVE4 cells, but not the OVE16 cells (Figure 3B). To test further the possible effects of TGFβ on enhancing stemness, the expression of several stem cell markers was assessed after 7 days of TGFβ treatment: *CD44*, *Sca-1*, *CD133*, *Oct3/4*, *Aldh1* and *Lgr5*. TGFβ induced a notable 5-fold increase in the levels of *CD44* mRNA, with a smaller increase in *Sca1* transcripts (Figure 3C). Western blot analysis showed that TGFβ increased CD44 protein levels within 24 hours in both OVE4 and OVE16 cells (Figure 3D).

**Figure 4:**
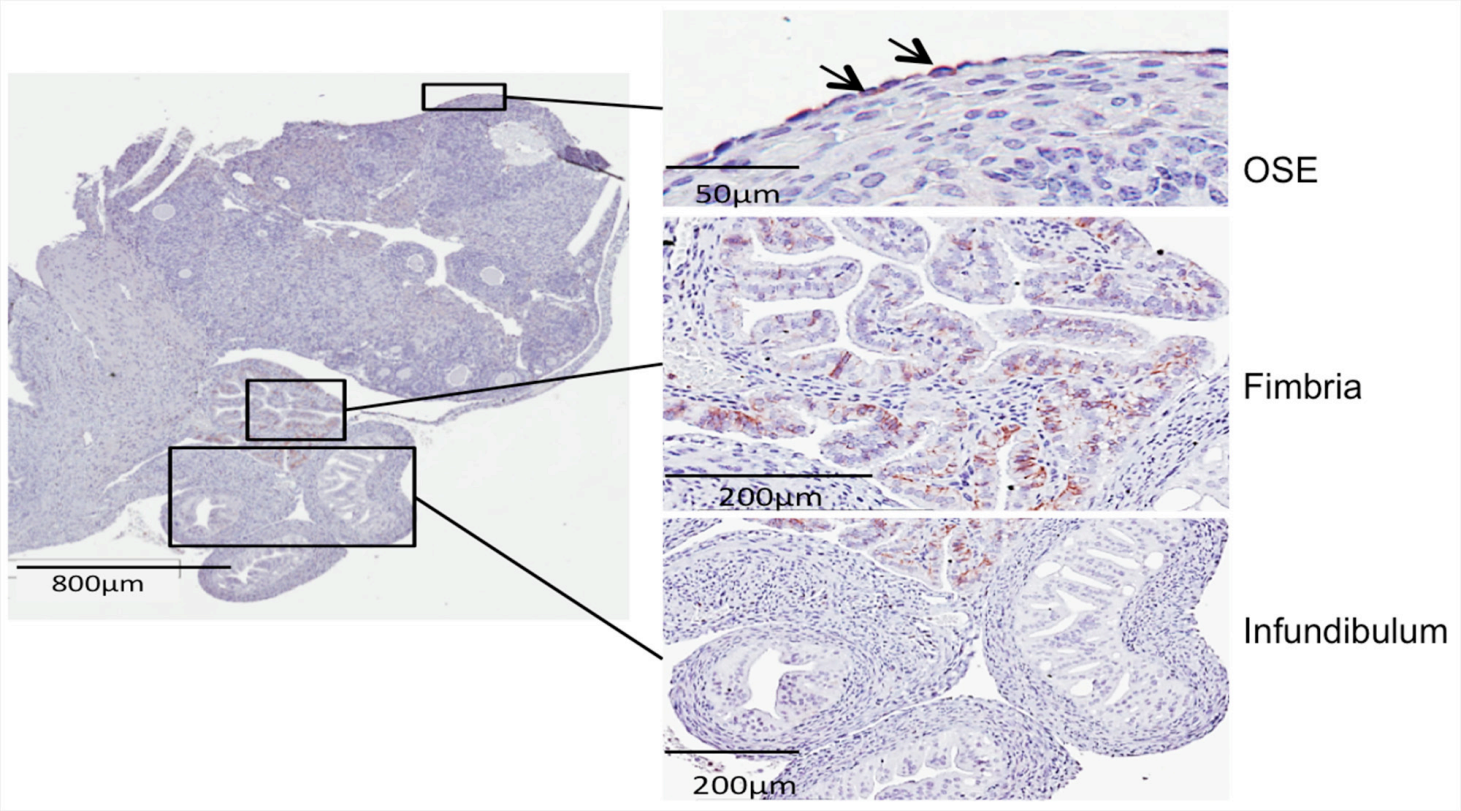
Immunohistochemistry shows CD44 staining only in the fimbriae and a few cells in the ovarian surface epithelium

When CD44 positive cells were enriched by fluorescence-activated cell sorting (FACS), they were able to form more spheres than CD44 negative cells in both OVE4 and OVE16 cell lines (Figure 3E). Further examination of CD44 abundance showed that TGFβ increases the fraction of CD44-expressing cells, as determined by flow cytometry using a pan-CD44 antibody (Supplementary Figure 2A) and by immunofluorescence (Supplementary Figure 2B). Immunohistochemistry was used to localize CD44 in mouse oviducts, and revealed staining only in the distal end of the fimbria, as well as in a few cells in the epithelium on the surface of the ovary (Figure 4).

### TGFβ suppresses PAX2 expression in OVE cells

**Figure 5:**
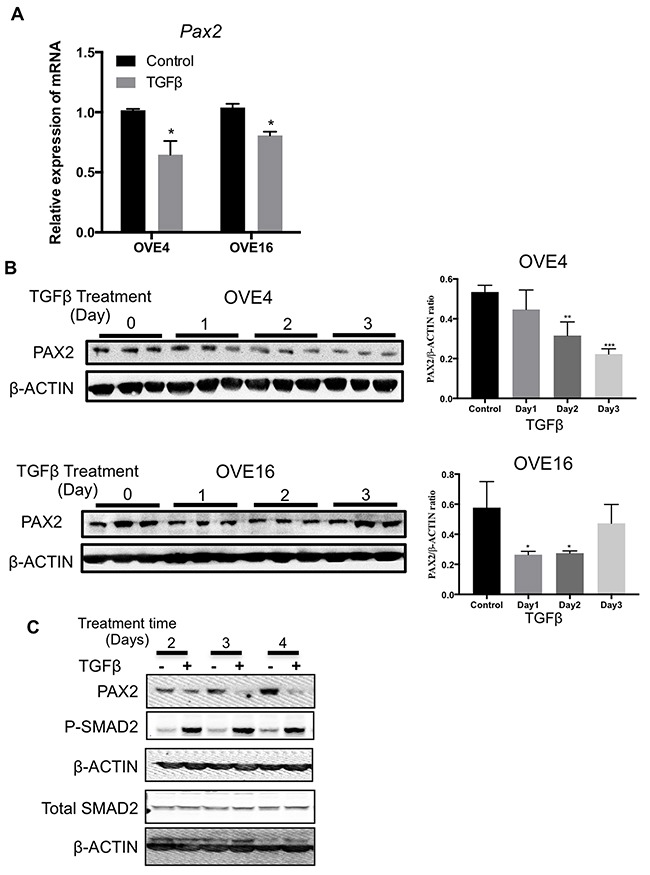
TGFβ suppresses *Pax2* expression in oviductal epithelial cells **(A)** qPCR analysis for mRNA encoding *Pax2* for OVE4 and OVE16 cells treated for one day with TGFβ. **(B)** Western blots and histogram of densitometric analysis show decreased abundance of PAX2 after 1-2 days of 10ng/ml TGFβ treatment of OVE4 and OVE16 cells. **(C)** Western blot shows time course of TGFβ treatment resulting in coincident reduction of PAX2 abundance with increased SMAD2 phosphorylation. All data are representative of three independent experiments except SMAD2 western blot that is representative of two independent experiments. Data presented in histograms are mean ± SEM. * p<0.05; ** p<0.01; *** p<0.001.

Treatment with TGFβ led to a significant inhibition of *Pax2* transcript levels in OVE cells as determined by qPCR (Figure 5A). PAX2 protein abundance was reduced by TGFβ in OVE4 and OVE16 cells within 1-2 days (Figure 5B), suggesting a possible inverse relationship between PAX2 levels and *CD44* and *Sca-1* expression. To more closely examine the time course of PAX2 repression by TGFβ, cells were treated with 10 ng/ml of TGFβ and the proteins were collected after 2 to 7 days. TGFβ treatment resulted in a reduction in PAX2 levels in OVE4 cells by day 2, and remained coincident with SMAD2 phosphorylation throughout the time course (Figure 5C and data not shown).

### PAX2 down-regulates *CD44* and *Sca-1* expression in OVE cells

**Figure 6:**
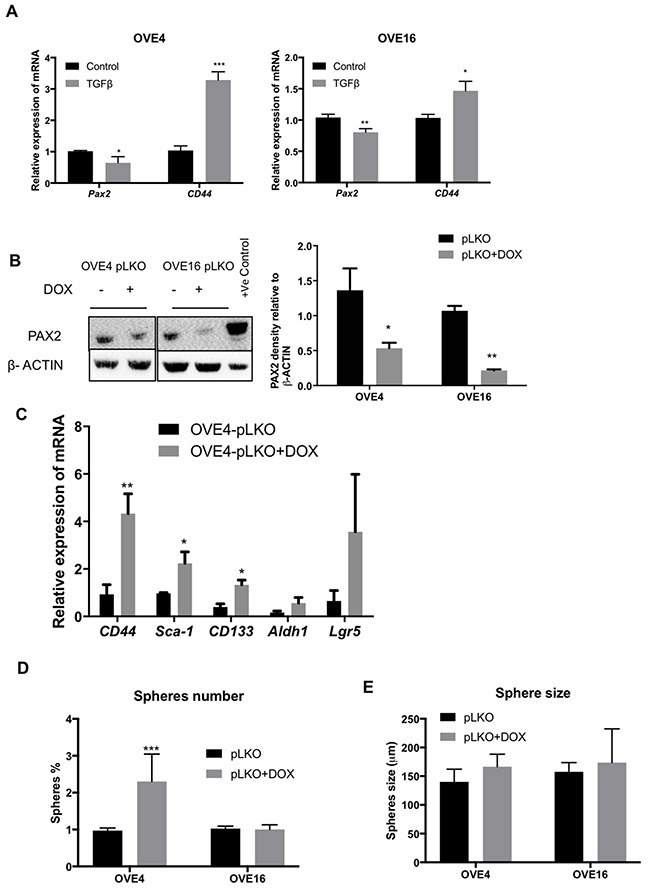
PAX2 down-regulates CD44 expression **(A)** qPCR analysis shows decreased levels of *Pax2* mRNA is associated with increased levels of *CD44* mRNA in OVE4 and OVE16 cells after 1 day of TGFβ treatment. **(B)** Western blot and densitometric analysis show PAX2 knockdown after doxycycline treatment of OVE cells transduced with *Pax2* shRNA constructs. **(C)** Knockdown of PAX2 increases mRNA for stem cell markers *CD44, Sca-1 and CD133*. **(D and E)** Sphere-forming capacity of OVE cells increased after PAX2 knockdown only in OVE4 cells, and there was no effect on sphere size. All experiments were performed at least three times and for experiments shown in B-E, samples were collected for analysis 4-6 cell passages after initiating doxycycline treatment. * p<0.05; ** p<0.01; *** p<0.001.

Thus far, the results indicate that TGFβ is able to suppress PAX2, down-regulate epithelial markers (*Cdh1*), up-regulate EMT markers (*Snai1* and *Zeb1*) and increase stem cell marker (*CD44* and *Sca1*) expression. The changes in both *CD44* and *Pax2* expression occur within one day of TGFβ treatment in both OVE4 and OVE16 cells (Figure 6A), suggesting a possible association. To determine whether PAX2 negatively regulates CD44 and SCA-1 and to better understand the consequences of the loss of PAX2 in fallopian tube epithelium, we knocked down PAX2 using doxycycline-inducible shRNA (pLKO) in OVE4 and OVE16 cells. PAX2 was decreased by 61% in OVE4-pLKO and 80% in OVE16-pLKO in doxycycline-treated cells compared to cells without doxycycline (Figure 6B). CD44 expression is normally very low in subconfluent cultures of OVE, but knockdown of PAX2 resulted in significant increases in transcripts for *CD44*, *Sca-1* and *CD133* mRNA (Figure 6C), with no significant changes in *Aldh1* and *Lgr5*. However, quantitative analysis of sphere formation by both OVE4-pLKO and OVE16-pLKO cells showed increased sphere-forming capacity after PAX2 knockdown only in OVE4-pLKO cells (Figure 6D) and no effect on sphere size in either cell line (Figure 6E).

### PAX2 overexpression decreases stemness in ovarian epithelial cells

**Figure 7:**
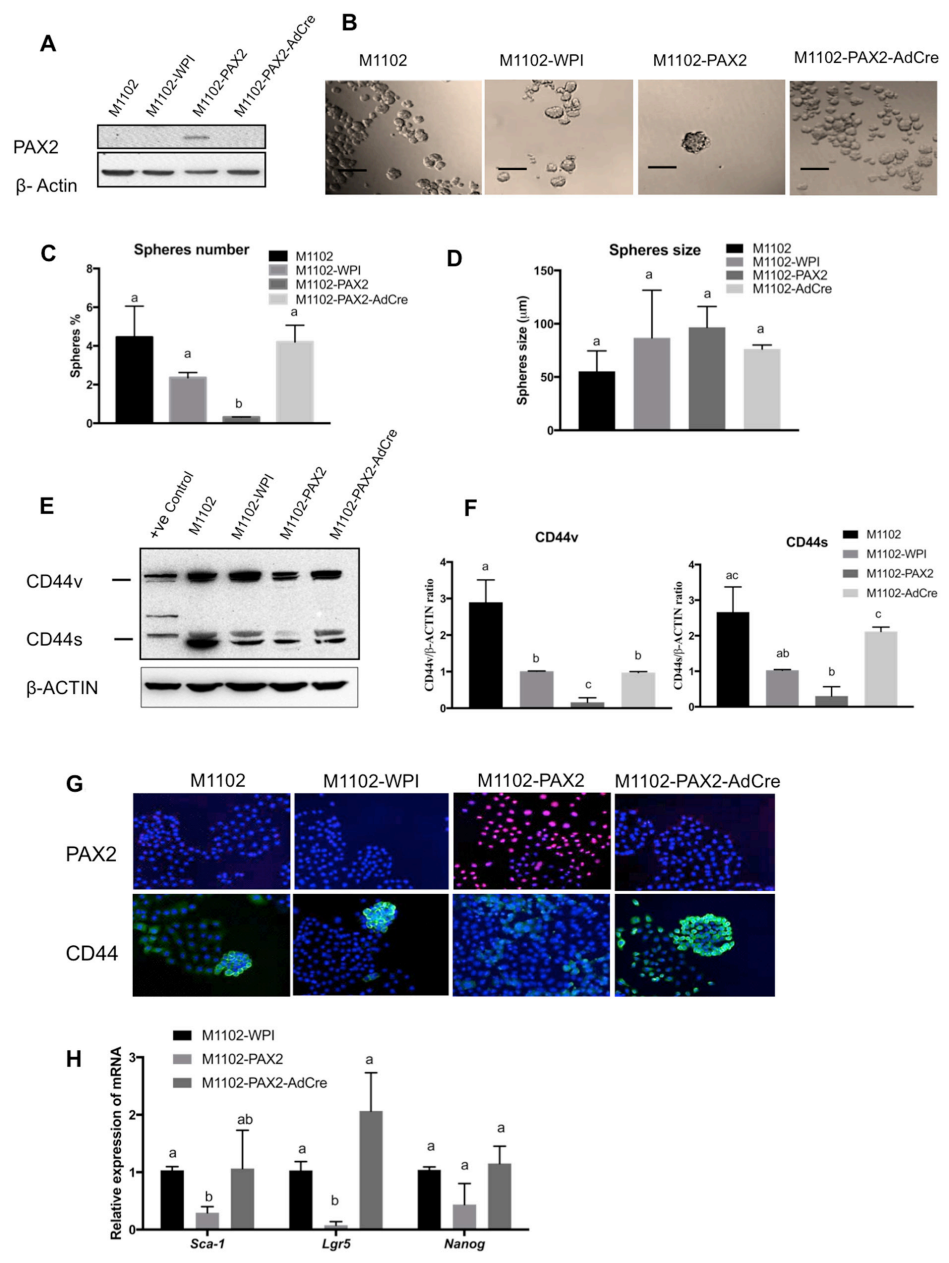
PAX2 decreases stem cell characteristics in mouse OSE cells (M1102) **(A)** Western blot shows the forced expression of PAX2 in M1102, with subsequent loss of expression in cells treated with adenovirus expressing Cre recombinase (AdCre) to delete the floxed PAX2 construct. **(B-D)** Overexpression of PAX2 in M1102 cells decreases sphere formation in suspension (N=3), but has no effect on sphere size. **(E)** Representative western blot shows that expression of PAX2 in M1102 cells decreases both variant and standard forms of CD44. **(F)** Densitometric analysis of CD44v and CD44s protein levels in M1102 cells from western blots normalized to β-actin (N=3). **(G)** Immunofluorescence staining shows that induction of *Pax2* expression in M1102 cells decreases abundance of CD44. **(H)** qPCR analysis for stem cell markers shows that expression of *Pax2* decreases mRNA encoding for *Sca-1* and *Lgr5* in M1102 cells (N=3). Data are presented as mean ± SEM. Scale bar in (B) indicates 100μm. Different letters denote values that are significantly different (p<0.05).

To better understand the role of PAX2 in inhibiting stemness, we forced *Pax2* expression in OSE cells (M1102) which do not normally express *Pax2*. We have previously shown that M1102 cells have a subpopulation with stem cell characteristics that include SCA-1 expression and sphere formation [31]. M1102 cells were transduced with a loxP-flanked construct expressing *Pax2* as shown by western blot (Figure 7A). In M1102 cells expressing PAX2, sphere formation was significantly reduced, compared to parental cells and the vector control. When the *Pax2* gene was deleted by treatment of the cells with adenovirus expressing Cre recombinase, the capacity for sphere formation was restored (Figure 7B and 7C). Sphere size was not affected by any of the treatments (Figure 7D). Levels of CD44 protein were reduced by *Pax2* expression in M1102 cells, as shown by western blot (Figure 7E and 7F) and immunofluorescence (Figure 7G). In addition, expression of *Pax2* decreased the expression of other stem cell markers such as *Sca-1* and *Lgr5* in M1102 cells (Figure 7H).

### PAX2 enhances cell differentiation in 3D cultures to form luminal structures

**Figure 8:**
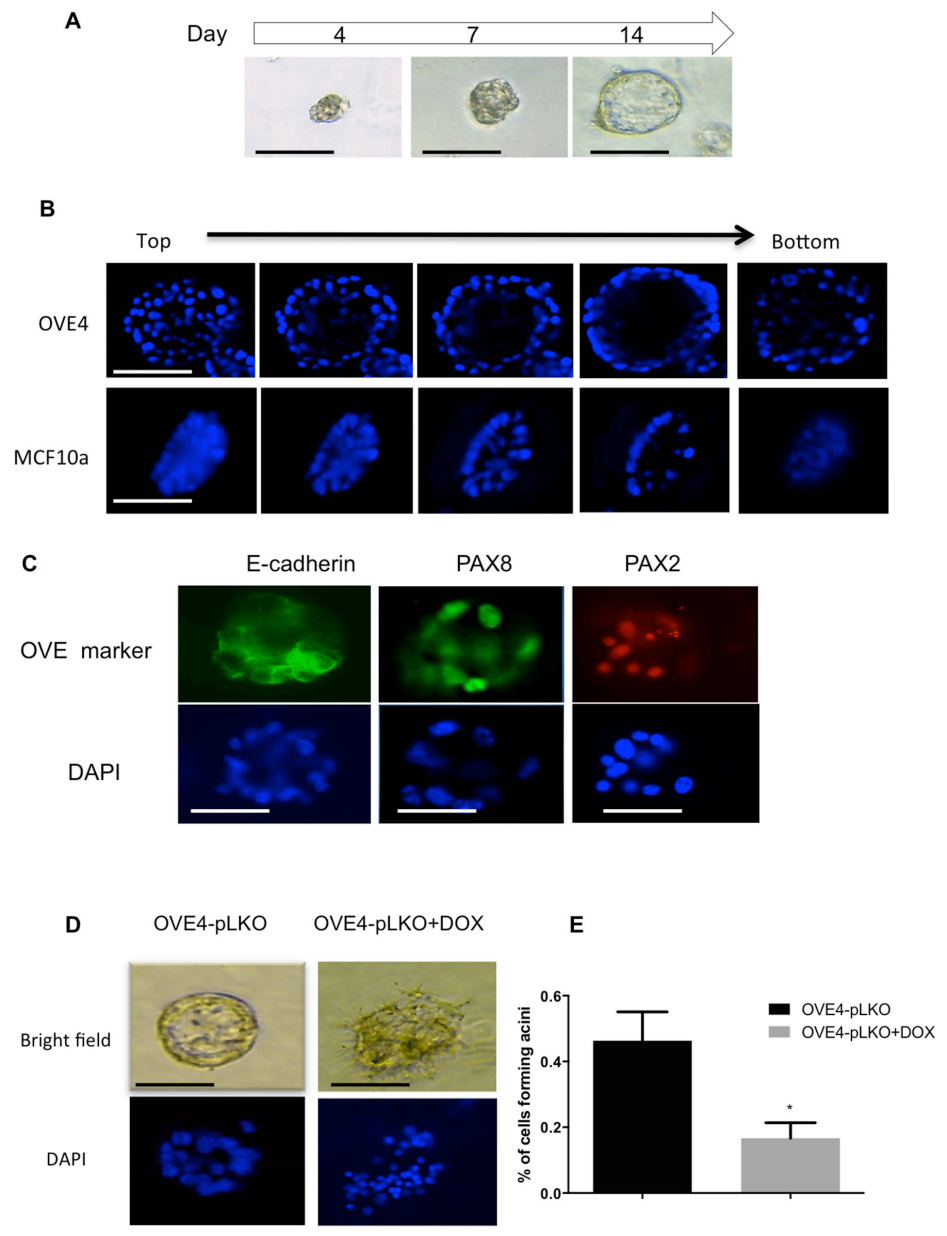
PAX2 is required for epithelial differentiation of OVE cells **(A)** Formation of luminal structure by OVE cells cultured in matrigel starts with small spheres by day 4 and ends with spheroids with a lumen by day 14. **(B)** Immunofluorescence by Z-stack imaging starting from the top of spheroids formed by OVE4 and MCF10a cells in matrigel after 14 days of culture shows the lumen in the middle of each spheroid. **(C)** OVE4-derived luminal structures express E-cadherin and the oviductal markers, PAX2 and PAX8, as detected by immunofluorescence. **(D)** Knockdown of PAX2 (right panels) disrupts the formation of luminal structures by OVE4 cells (left panels), which is quantified in **(E)**. Data are presented as mean ± SEM (N=3). Scale bars in (A-C) indicate 100μm. * indicates p<0.05.

To determine whether PAX2 has an essential role in maintaining the epithelial differentiation of OVE cells, OVE cells were cultured in matrigel using methods previously reported for mammary epithelial cells [36]. After 14 days in 3D culture, OVE4 cells, which express relatively high levels of PAX2, formed polarized structures with a lumen (Figure 8A) that resemble the acini formed by MCF10A breast epithelial cells (Figure 8B). The luminal structures formed by OVE4 cells have E-cadherin localized to the membrane and PAX8 and PAX2 in the nuclei (Figure 8C). OVE16 cells formed solid spheres without a lumen (Supplementary Figure 3). To confirm the role of PAX2 in the formation of these luminal structures, we assessed OVE4-pLKO cells after PAX2 knockdown with doxycycline. Both epithelial cell organization and lumen formation were disrupted (Figure 8D), reducing significantly the number of luminal structures formed after PAX2 knockdown (Figure 8E).

## DISCUSSION

A growing number of studies have suggested that STICs at the distal end of the fallopian tube are a precursor lesion for ovarian carcinomas [12, 37, 38]. Multiple gene mutations and alterations have been investigated to identify promising biomarkers for STICs, as reviewed by Mehra et al. (2011) [39]. STICs have been characterized by loss of PAX2 [12, 25] and expansion of CD44-expressing cells [22]; however, the mechanisms contributing to these changes are unknown. The number of lifetime ovulations is an established risk factor for ovarian cancer [40], and fallopian tube lesions may be increased by the repeated process of ovulation [32]. Both the ovarian surface and adjacent fimbria are exposed to the ovulatory follicular fluid containing inflammatory cytokines [41], creating a pro-inflammatory microenvironment that could be suitable for promoting stem-like properties [42]. Since the epithelial cells that line the fimbria are exposed to follicular fluid during ovulation, we explored the possibility that TGFβ, a component of follicular fluid, may influence the OVE cells as it does the OSE cells of the ovary [31].

The morphology of fallopian tube epithelial cells has been reported to change *in situ* depending on the phase of the menstrual cycle, and notably can become elongated and pseudostratified, a change that may be due to the changing profile and/or abundance of hormones or cytokines released by the epithelial cells and/or present in the follicular fluid [43]. Thus, we examined the effect of TGFβ as an inflammatory cytokine and report here its ability to induce EMT in OVE cells as shown by changes in morphology, loss of E-cadherin expression, increased SNAIL and Vimentin, and enhanced motility.

Interestingly, induction of EMT in OVE cells by TGFβ was also accompanied by increased expression of the stem cell markers CD44 and, to a lesser extent, SCA-1, as well as increased sphere size in a sphere forming assay of OVE4 cells. Despite the fact that TGFβ significantly inhibited proliferation of OVE cells when cultured as a monolayer, the larger spheres that were produced when OVE4 cells were cultured in suspension suggests that TGFβ activates alternative pathways in non-attached cells to increase OVE cell proliferation, resulting in an increase in sphere size, rather than increasing sphere number. A recent publication has shown that ovarian cancer cells in suspension acquire an EMT phenotype [44], so it is possible that inducing an EMT phenotype in the OVE cells enhanced their ability to proliferate in suspension and that this action is independent of any effects on PAX2. This is in contrast to the effects of TGFβ on OSE sphere formation, which results in suppression of growth in cells cultured both in monolayer and in suspension, but increases sphere number and has similar actions to increase SCA-1 expression [31].

The strong up-regulation of CD44 in OVE cells treated with TGFβ may relate to putative stem cells in the fallopian tube. In the fimbria, basal cells extracted from human fallopian tubes express CD44 and are able to self-renew and form spheres [22]. We therefore focused on this stem cell marker for further study, attempting to identify possible stem-like cells in the OVE cell lines. OVE cells visualized by immunofluorescence after TGFβ treatment showed a higher intensity of CD44 staining, which was supported by increased expression as seen in western blots. CD44 is expressed in various isoforms whose unique functions and relevance to tumor initiation are not yet fully defined [45]. OVE cells have low expression of the variant form of CD44, which was increased by TGFß treatment. These results suggest that exposure to TGFβ, perhaps from ovulatory follicular fluid or prolonged inflammation, can promote changes in OVE cells that are similar to those identified in STIC formation.

One of the most interesting observations in this study is that TGFβ significantly decreased PAX2, suggesting that PAX2 opposes TGFβ action on the state of differentiation of OVE cells. TGFβ has been reported to suppress PAX2 protein levels in dysplastic kidney cells [46]. While the mechanism is still unclear, a chromatin immunoprecipitation assay on HEK293 cells treated with TGFβ using anti-SMAD2/3 antibody indicated that TGFβ signaling can lead to direct binding of SMAD2/3 to the PAX2 promoter [47], although this work is not yet published. This supports our observation that repression of PAX2 is coincident with increased SMAD2 phosphorylation, suggesting possible regulation by the canonical TGFβ signaling pathway. Since loss of PAX2 expression is a primary characteristic of STICs, our observations suggest a possible role for TGFβ in inducing STIC formation. The mechanisms that restrict or promote STIC formation have not yet been elucidated; some studies suggest that these structures develop from SCOUTs that have lost PAX2 expression [12]. In a recent study, some types of SCOUTs were shown to acquire a stem cell phenotype by expressing ALDH1 [25], while another study has reported that STICs have an expanded number of CD44-positive cells compared to normal tubal epithelium [22]. Our results indicate that PAX2 can negatively regulate *CD44* and *Sca-1* expression in both OVE cells and OSE cells and also influences the capacity for sphere formation, both characteristics of stem cells. PAX2 therefore appears to preserve the epithelial differentiation of OVE cells, and loss of its expression, for example during EMT, is associated with a transition to a more stem-like state. Potentially, the frequent loss of PAX2 in SCOUTs, STICs and high-grade serous ovarian cancers is essential to allow for the stem cell-like plasticity required for tumor progression. Interestingly, ectopic expression of PAX2 in the STOSE model of high-grade serous ovarian cancer led to delayed tumor formation and reduced dissemination [48].

Few CD44+ cells were found in the layer of epithelial cells on the ovary, but they were abundant in the distal end of the oviduct, the site of origin of most STICs. CD44 was localized primarily in the basal cells, but it remains to be determined whether PAX2 inhibits CD44 expression in oviductal cells *in vivo*, consistent with the regulation that we demonstrated *in vitro*.

PAX2 has a well-established role in the epithelial differentiation of the embryonic reproductive tract [49], and our results suggest that PAX2 continues to promote this more differentiated state in adult epithelia. Surprisingly, OVE cells have the ability to form luminal structures that resemble the acini formed by breast epithelial cells. While this architecture resembles the breast acini, it also resembles the normal oviduct, including the expression of PAX2 and PAX8. The formation of these luminal structures was dependent on the presence of PAX2. PAX2 knockdown in OVE4 cells did not prevent initial formation of spheres in the matrigel during the first four days, but these spheres were disrupted from further progression in both growth and lumen formation. At this time the mechanisms by which PAX2 promotes the organization of this polarized, luminal structure is unknown, but its reported ability to promote epithelial differentiation during embryo development [13, 14], and to transactivate the Wilm's tumor suppressor [50] provide promising avenues for investigation. Interestingly, OVE16 were unable to make luminal structures, perhaps due their lower expression of E-cadherin. Further studies are needed to precisely define signaling changes induced by PAX2 that contribute to the biology of oviductal cells and the transition to SCOUTs, STICs and early ovarian cancer.

## MATERIALS AND METHODS

### Cell lines and cell culture

Human embryonic kidney 293T cells (ATCC, Manassas, VA) were maintained in Dulbecco's modified Eagle's medium (Life Technologies Inc., Burlington, ON) supplemented with 10% fetal bovine serum (PAA Laboratories, Pasching, Austria). Three clonal OVE cell lines (OVE4, OVE16 and OVE22) were established for this study. Twenty-five day old female FVB/N mice (Jackson Laboratory, Bar Harbor, ME) were housed with free access to food and water and euthanized in accordance with the Canadian Council on Animal Care's *Guidelines for the Care and Use of Animals* under a protocol approved by the University of Ottawa's Animal Care Committee. Mice oviducts were isolated, washed with phosphate-buffered saline (PBS), cut into fragments and minced on tissue culture plates. Tissue fragments were incubated in 0.25% trypsin (Invitrogen, Carlsbad, CA) at 37C° and 5% CO_2_ for 30 min to dissociate the OVE cells. Small tissue fragments with dissociated cells were cultured in minimal essential media (MEM; GE Healthcare, Little Chalfont, UK) supplemented with 4% fetal bovine serum, 0.01 nM of estrogen (Sigma-Aldrich, St. Louis, MO), 5 U/ml of penicillin and streptomycin solution (Penstrep; Sigma-Aldrich), 0.1 μg/ml of gentamicin (Invitrogen), 0.02 μg/ml of epidermal growth factor (EGF; R&D Systems, Minneapolis, MN) and 1 μg/ml of insulin-transferrin-sodium-selenite supplement (ITSS; Roche, Basel, Swizerland) for two weeks or until the cells attached and spread out. The small fragments were washed off and only attached cells were trypsinized, re-plated as single cells and subsequent colonies were isolated and grown to establish independent lines. The epithelial nature of the resulting cells was verified by analyzing E-cadherin, CK19 and PAX8 expression. M1102 cells are primary cultures of mouse ovarian surface epithelial cells and were maintained in MOSE media as described previously [31]. When indicated, OVE cells were treated with 10 ng/ml human TGFβ1 (R&D Systems) and refreshed every three days until the end of the experiment.

### Lentiviral vectors

The murine *Pax2* cDNA, corresponding to pax2-b variant, was cloned from the murine oviduct and inserted into pWPI (Addgene plasmid #12254) to generate the lentivirus expression vector (WPI-Pax2-IRES-eGFP), as described previously [48]. For shRNA mediated knockdown, lentiviral vectors Tet-pLKO-neo and Tet-pLKO-puro were used to generate PAX2 specific knockdown vectors pLKO-17 (CCCAAAGTGGTGGACAAGATT) and pLKO19 (CAGGCATCAGAGCACATCAAA), respectively. *Pax2* target sequences for each vector are indicated in brackets. Tet-pLKO-neo (Addgene plasmid 21916) and Tet-pLKO-puro (Addgene plasmid 21915) were gifts from Dmitri Wiederschain [51]. Lentiviral vectors were prepared by co-transfection of vector plasmids, with packaging plasmid pCMVR8.74 (Addgene plasmid #22036) and the ecotropic envelope expression plasmid, pCAG-Eco (Addgene plasmid 35617) into 293T cells as described previously [52]. Plasmids pWPI and pCMVR8.74 were gifts from Didier Trono and plasmid pCAG-Eco was a gift from Arthur Nienhuis and Patrick Salmon.

### PAX2 overexpression and knockdown

M1102 cells were infected with either the control lentiviral vector, WPI, or WPI-Pax2-IRES-eGFP to generate cell lines M1102-WPI and M1102-PAX2, respectively. As an additional control, the *Pax2* expression cassette was deleted from M1102-PAX2 using Cre recombinase delivered by transient infection with adenovirus expressing Cre recombinase, as described previously [48]. For PAX2 knockdown, OVE4 and OVE16 cells were infected with lentiviral vectors (pLKO17, pLKO19) expressing doxycycline-inducible shRNAs targeting murine *Pax2*. Cells infected with a control lentivirus (Tet-pLKO-puro) were used as a negative control. G418 and puromycin were used to select cells with stable incorporation of the Pax2 or control knockdown vectors. In all experiments including cells with knockdown of *Pax2*, cells were cultured in the presence of doxycycline throughout the experiment.

### Proliferation assay

Cells were plated in 10 cm dishes at a density of 1×10^4^ in OVE media, described in the cell culture section. TGFβ (10 ng/ml) was added at the time of plating cells. The media were replaced with fresh media with TGFβ every three days. TGFβ-treated and control cells were trypsinized after seven days of TGFβ treatment and cells were counted using Vi-CELL XR Cell Viability Analyzer (Beckman Coulter, Inc).

### Migration assay

Capacity for cell migration was assessed using the scratch-wound-healing assay to measure the effect of TGFβ treatment on OVE cells. The scratch-wound-healing assay was performed using a mechanical tool to create a wound in a confluent cell culture. Detached cells were washed with PBS and fresh OVE medium was added. To assess the effect of TGFβ treatment, OVE cells were pre-treated with and without 10 ng/ml TGFβ for three days before the migration assay and after creating the scratch. The cells were incubated at 37C° and images were taken at 0 and 24 hours for migratory cells moved towards the wound. The gap closure analysis was done using ImageJ software (NIH Image).

### Gene expression analysis

Gene expression in OVE cells was analyzed by qPCR. RNA was extracted using RNeasy Mini Kits (Qiagen, Valencia, CA) following the manufacturer's instructions. cDNA was made using Reverse Transcription kit (Qiagen). Gene amplification was determined by qPCR using either Fast SYBR Green Master Mix (Invitrogen) for primers or *iTaq* universal probes supermix (Bio-Rad, Hercules, CA) for probes. The real-time thermal cycler program was 95C° for 10 min, followed by 40 cycles of 95 C° for 10 sec and 60 C° for 30 sec. Primer pairs and probes are provided in Supplementary Table 1. The gene expression levels were analyzed relative to the endogenous control *Ppia* (Invitrogen) for primers and *TBP* (Integrated DNA Technologies, Coralville, IA) for the probes.

### Sphere formation assay

OVE cells were trypsinized and single cells were passed through a 40 μm cell strainer. Single cells (5000) were cultured in suspension in low attachment plates for two weeks using DMEM/F12 media (Sigma-Aldrich) containing 0.3% FBS, B27 supplement 50X (0.02 mg/ml; Life Technologies, USA), 0.2 μg/ml of EGF, 0.4 μg/ml of mouse fibroblast growth factor (FGF; R&D Systems), 4 μg/ml of heparin, 10 U/ml Penstrep and 0.2 μg/ml of gentamicin. The ability of the OVE and M1102 cells to form spheres was assessed by measuring sphere diameters and counting the spheres with diameters more than 50μm. The percentage of cells that formed spheres was determined and the average diameter of the spheres was measured using ImageJ software (NIH Image). Sphere number and size were determined in at least three independent experiments, each performed in triplicate wells.

### Flow cytometry for CD44 expression

OVE cells were dissociated using non-enzymatic stripper (MULTICELL Cell Stripper) and the cells passed through a 40 μm cell strainer. Single cells were incubated with antibodies to human/mouse CD44 conjugated to APC (1:5000; eBioscience, San Diego, CA) for 15 minutes at 4C°. Unbound antibody was removed with washing buffer and the fraction of cells with surface protein labeled with CD44 antibody was determined using a MoFlo cell sorter (Dako Cytomation).

### Western blot analysis

Cellular proteins were recovered from cell cultures using the M-PER mammalian protein extraction reagent (Thermo Fisher, Waltham, MA). Cell proteins were separated and transferred to nitrocellulose as previously described [53], and then probed with the following antibodies: anti-mouse PAX2 (1:20000, Santa Cruz Biotechnology, Dallas, Texas) and anti-rabbit CD44 (1:500), anti-rabbit E-cadherin (1:10,000), anti-rabbit vimentin (1:10,000), anti-rabbit Snail (1:10,000), anti-rabbit cytokeratin 19, anti- rabbit OVGP (1:1000) (all from Abcam, Cambridge, UK). Anti-rabbit (1:10,000, Abcam) or anti-mouse (1:10,000, Sigma-Aldrich) horseradish peroxidase-conjugated antibodies were used as secondary antibodies. Immunoreactive proteins were detected using chemiluminescence (Clarity Western ECL Substrate; Bio Rad) and the blots were imaged using FluorChem FC2 (Alpha Innotech). The densitometric analyses of protein abundance were performed using ImageJ software (NIH Image).

### Immunofluorescence

Immunofluorescence was performed on cells grown on coverslips and in matrigel. The cells were fixed with 4% paraformaldehyde (Sigma-Aldrich) for 20 min and permeabilized with 0.5% Triton X-100 for 30 min for cells grown on coverslips or 0.2% Triton X-100 for 10 min for cells grown in matrigel. After blocking in 5% goat serum, cells were probed with each antibody as follows: anti-mouse PAX2 (1:500, Santa Cruz), anti-mouse PAX8 (1:200, Santa Cruz), anti-rabbit E-cadherin (1:200, Abcam) and anti-rat CD44 (1:200, Abcam). Alexa Fluor goat anti-mouse IgG (1:1000), goat anti-rabbit IgG (1:500) and goat anti-rat (1:500; all from Molecular Probes, Carlsbad, CA) were used as secondary antibodies. The cells on coverslips were then mounted to microscope slides using Vectashield hard set mounting medium with DAPI (Vector Laboratories, Burlingame, CA) and the immunofluorescence images were visualized and analyzed using an inverted fluorescence microscope (Axioskop 2 MOT plus, Zeiss) and Axiovision software.

### Immunohistochemistry (IHC)

Reproductive tracts from FVB/N mice were extracted and fixed in formalin overnight, then transferred to 70% ethanol. The tissue was paraffin-embedded, cut in 4 μm sections, de-paraffinized and rehydrated in graded ethanol. For antigen retrieval, the tissues were heated in a microwave in antigen unmasking solution (Vector laboratories). For regular IHC, tissue sections were blocked with DAKO protein block serum-free (DAKO, Glostrup, Denmark) for 1 hour and then incubated overnight at 4C° with anti-rat CD44 (1:400, Abcam). After washing in PBS, the sections were incubated with ImmPRESS anti-rat secondary antibody (Vector Laboratories). The immunoreactivity was imaged using Aperio ScanScope system and ImageScope software (Aperio).

### 3D culture in matrigel

Chamber slides (8 wells; Thermo Fisher) were covered with 60μl of cold growth factor-reduced matrigel (Trevigen, Gaithersburg, MD) and incubated at 37C° for 30 minutes to polymerize the matrigel. Cells were mixed with assay media (MEGM™ Mammary Epithelial Cell Growth Medium supplemented with 5% fetal bovine serum, 1 ng/ml EGF and 0.5 ng/ml of insulin, hydrocortisone, cholera toxin and gentamicin (all from lonza, Basel, Swizerland) and plated on top of the matrigel as described previously [36]. The cells were covered with assay media containing 4% matrigel and incubated at 37°C for 14 days.

### Statistical analyses

All experiments were performed a minimum of three times. Statistical analyses were performed using GraphPad Prism software (GraphPad, La Jolla, CA). A Student t-test (two groups) or an ANOVA with a Tukey post-test (multiple groups) were used to determine statistical significance (P< 0.05). In two-group analyses containing multiple tests (eg. Figure 2C), multiple hypothesis tests were controlled for with the Benjamini-Hochberg False Detection Rate (FDR) method, setting a threshold FDR of 5%. Error bars represent the standard error of the mean.

## SUPPLEMENTARY MATERIALS FIGURES AND TABLE


