# Supplementary material for: PAX2 maintains the differentiation of mouse oviductal epithelium and inhibits the transition to a stem cell-like state
Source: Oncotarget. 2017 Aug 10;8(44):76881–97. doi: 10.18632/oncotarget.20173 (PMC5652750; doi:10.18632/oncotarget.20173)
Supplement: Supplementary file 1 [file oncotarget-08-76881-s001.pdf]

PAX2 maintains the differentiation of mouse oviductal epithelium and inhibits the transition to a stem cell-like state

SUPPLEMENTARY MATERIALS

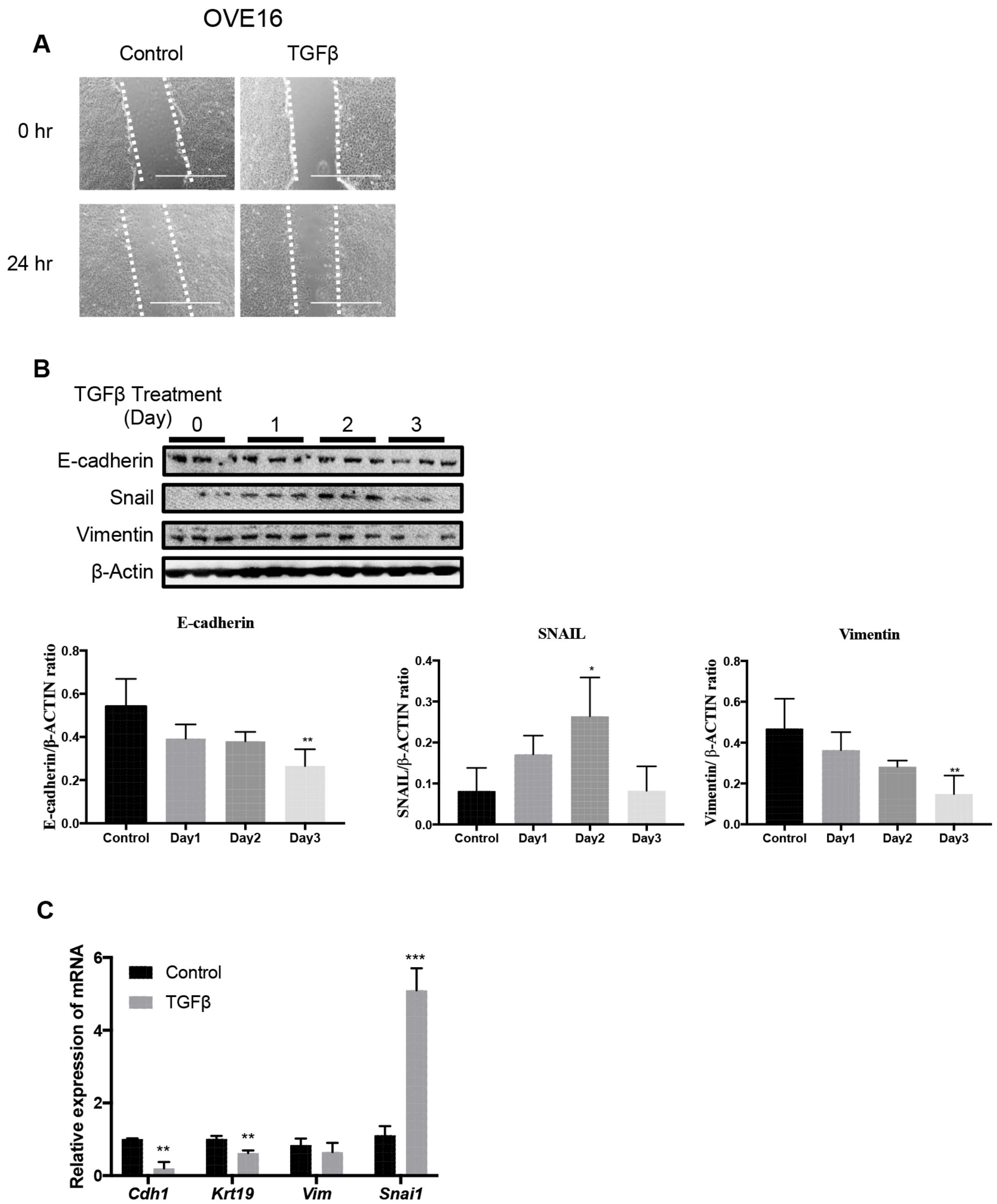

Supplementary Figure 1: TGFβ induces EMT in OVE16 oviductal epithelial cells. (A) TGFβ pre-treatment for 3 days did not increase cell migration of OVE16 cells, as seen 24 hours after wounding. **(B)** Western blot and densitometric analysis for epithelial and EMT proteins (E-cadherin, SNAIL, Vimentin) in OVE 16 cells treated with TGFβ for 3 days. **(C)** qPCR analysis for mRNA encoding for epithelial and EMT genes for OVE16 cells treated for one day with TGFβ. Data are from three independent experiments. Data presented in histograms are mean ± SEM. Scale bars indicate 400µm. * indicates $p<0.05$; ** indicates $p<0.01$.

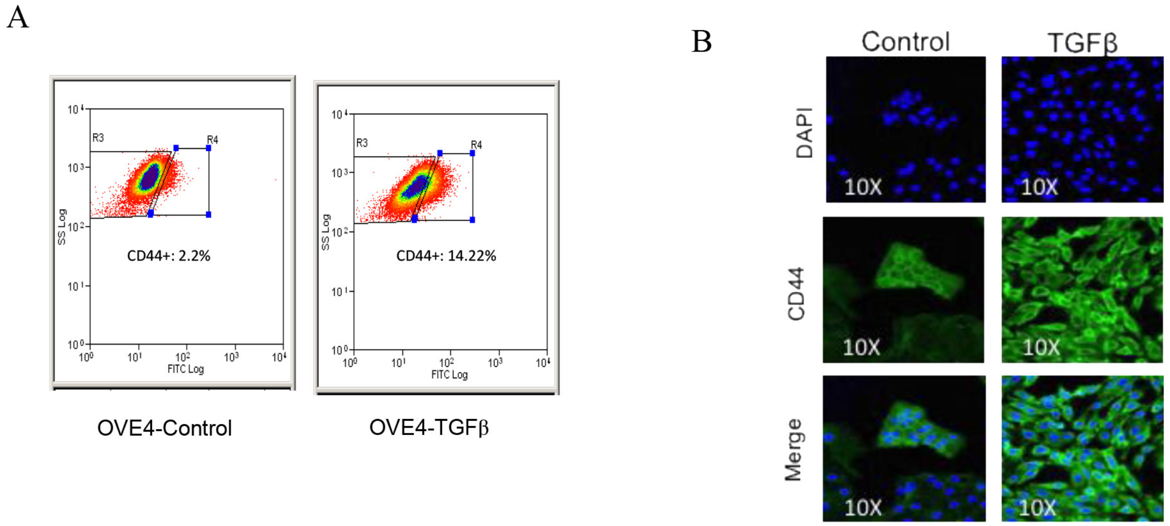

Supplementary Figure 2: (A) Flow cytometric analysis shows an increased percentage of OVE4 expressing CD44 after 7 days of TGFβ treatment. **(B)** Immunofluorescence microscopy reveals the presence of intense staining for CD44 in OVE4 cells after TGFβ treatment.

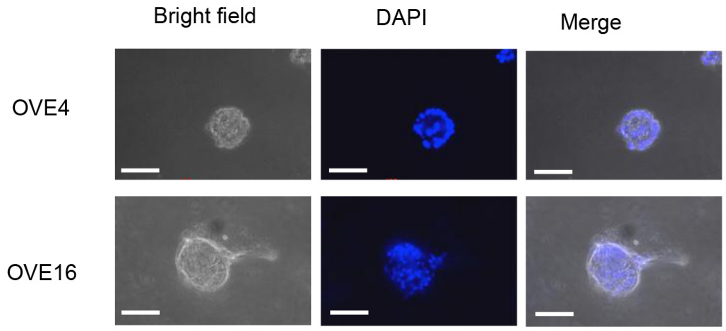

Supplementary Figure 3: Only OVE4 cells in matrigel form acini- like structure, whereas OVE16 cells form solid spheres. Scale bar is 100μm.

Supplementary Table 1 : List of primers and probes used for qPCR analysis

Gene	Sequence	Source
Cdh1	(Forward [F], 5'-atcctcgccctgctgatt-3'; Reverse [R], 5'-accaccgttctcctccgta-3')	Invitrogen
Vimentin	(Forward [F], 5'-cgccaggccaagcaggagtc-3'; Reverse [R], 5'-cgctccagggactcgttagtgc-3')	Invitrogen
Snai1	(Forward [F], 5'- gtctgcacgacctgtggaa-3'; Reverse [R], 5'- caggagaatggcttctcacc-3'),	Invitrogen
Pax2	(Forward [F], 5'-GGCATCTGCGATAATGACACA-3'; Reverse [R], 5'-GTGGAAAGGCTGCTGAACTT-3'), (5'-/56-FAM/TCCCCTGTT/ZEN/CTGATTTGATGTGCT CTG/3IABkFQ//-3')	Invitrogen Integrated DNA technology (IDT)
Pax8	(Forward [F], 5'-GCAGCTATGCCTCTGCTA-3'; Reverse [R], 5'-GCTGTAGGCATTGCCAGAAT-3').	Invitrogen
Ppia	(forward [F], 5'- AGGGTGGTGACTTTACACGC-3'; Reverse [R], 5'- GATGCCAGGACCTGTATGCT-3')	Invitrogen
Sca-1	(5'-/56-FAM/ATCTTTGCT/ZEN/ TACCCATCTGCCCTCC/3IABkFQ/-3'),	IDT
CD44	(5'-/56-FAM/TCTTCTGCC/ZEN/ CACACCTTCTCCTACT/3IABkFQ/-3'),	IDT
Aldh1	(5'-/56-FAM/AGTTAACCC/ZEN/ ACACCACCCCAGC/3IABkFQ/-3'),	IDT
Lgr5	(5'-/56-FAM/AGCTACCCG/ZEN/ CCAGTCTCCTACAT/3IABkFQ/-3'),	IDT
CD133	(5'-/ /56-FAM/CCG ATG CCA /ZEN/TCC AGG TCT GAG AA/3IABkFQ/-3'),	IDT
OcT3/4	(ID # Mm03053917_g1)	IDT
Pax2	(5'-/56-FAM/TCCCCTGTT/ZEN/CTGATTTGATGTGCT CTG/3IABkFQ//-3')	IDT
TBP	(5'-/56-FAM/ACTTGACCT/ZEN/AAAGACCATTGCAC TTCGT/3IABkFQ/-3')	IDT
